# Development of a User‐Centred Chronic Care Model for Patients With Heart Failure in a Limited‐Resource Setting: A Codesign Study

**DOI:** 10.1111/hex.70142

**Published:** 2025-01-05

**Authors:** Apinya Koontalay, Mari Botti, Anastasia Hutchinson

**Affiliations:** ^1^ School of Nursing and Midwifery Deakin University Victoria Australia; ^2^ Center for Quality and Patient Safety Research – Epworth HealthCare Partnership, Institute for Health Transformation Deakin University Geelong Victoria Australia

**Keywords:** codesign, design thinking, health services research, heart failure, participatory research, patient participation, stakeholder participation, user involvement

## Abstract

**Background:**

Health service leaders in Thailand face substantial challenges in addressing the needs of a growing population of patients with moderate to severe Chronic Heart Failure (CHF) who require acute care management and ongoing supportive care in the community. The large number of CHF patients requiring readmission for high‐level care places a significant burden on healthcare services.

**Methods:**

The design thinking model proposed by the Hasso‐Plattner Institute of Design at Stanford University underpinned an approach to developing a co‐designed, tailored, culturally acceptable model of chronic care for people with CHF. One consumer, 16 clinicians, and two organisational leaders participated in a codesign workshop that included three activities. The purpose of each activity was to (i) define the problem, (ii) brainstorm possible solutions and (iii) develop a prototype solution. The codesign workshop was one phase of a four‐phase codesign project. Data collected included physical data such as sticky notes and storyboards and audio recordings of codesign group discussions. Data were analyses using content analysis.

**Results:**

Nine prototype storyboards aimed at enhancing continuity of care for CHF patients emerged from the workshop activities. The proposed solutions focused on improving consumer access to evidence‐based information, multidisciplinary expertise and ongoing community support. Participants discussed and evaluated the viability and feasibility of each prototype before reaching a final decision on an optimal model. The preferred model was a nurse‐led case management service supported by a multidisciplinary team.

**Conclusion:**

Key stakeholders identified the importance of moving from a short‐term model of care to an integrated, multidisciplinary approach to providing long‐term support in the community. The final agreed prototype of a CHF Nurse Case Management service supported by a multidisciplinary team with a focus on community outreach addressed the key concerns of participants and was considered a feasible approach to developing a CHF chronic care service for the community in urban Bangkok, Thailand.

**Patient or Public Contribution:**

The process of codesign involved the engagement and participation of individuals with CHF, clinicians and organisational leaders throughout the research process.

## 1 Introduction

Chronic Heart Failure (CHF) contributes to morbidity, mortality and poor quality of life worldwide [1]. CHF is a growing burden on economies and healthcare systems as it is the cause of frequent acute care admissions and readmissions associated with high hospitalisation costs [1, 2]. It is a complex condition characterised by variations in symptom severity and intermittent episodes of deterioration that can be difficult to predict and manage effectively [3]. Patients with CHF and their families can face numerous challenges following hospital discharge related to limited knowledge about CHF medical management, the risk factors for the exacerbation of heart failure and required lifestyle changes [4]. Poor management causes substantial worsening of symptoms, often requiring hospital readmission [3]. Thus, patients with CHF need ongoing support in managing and maintaining this complex condition with treatment and support strategies that bridge the gap between acute hospital‐based and community care [5, 6]. In low to middle‐income countries (LMIC), the level of ongoing disease management support is highly variable and often suboptimal [7].

Despite the steadily rising burden of Non‐Communicable Diseases (NCD) in LMICs, limitations in health funding means only 1% goes towards prevention and management of these conditions [8]. Most LMICs struggle to provide health systems that deliver healthcare interventions to support people with NCD [9]. People with NCD need long‐term disease management support to make sustainable lifestyle adaptations that is provided through integrated and coordinated care between acute care, primary care settings and the community [10, 11]. Thailand is an upper middle‐income country that faces challenges in delivering sustainable approaches to NCD management and struggles with healthcare system resources and funding [10]. Chronic heart failure is an NCD that is increasing in prevalence and burden in Southeast Asia (SE‐Asia) and requires improvements in coordination of care [10, 12, 13].

Limitations of funding and resources are important considerations when translating international best practice guidelines into LMICs [9]. The majority of chronic disease self‐management interventions have been introduced and evaluated in high‐income countries (HICs), highlighting a gap in addressing the urgent healthcare needs of CHF patients in LMICs in regions such as SE‐Asia [14]. This gap presents a significant challenge as healthcare systems and resources in these settings may differ considerably from those in HICs [7]. Chronic disease self‐management programs established as effective in HICs may not be feasible in LMICs with a limited health workforce and resources [15], nor may they be culturally acceptable [4]. Many LMICs are facing a shortage of specialty trained health workers due to under‐investment in health education and migration of healthcare professionals to fulfill workforce demands in high‐income countries [16]. Each culture possesses distinct values, beliefs and behaviors that influence the understanding of advanced heart failure. Findings of a systematic review of the influence of culture in understanding of advanced heart failure found that patients from non‐Western cultures aimed to manage their condition and seek care when it aligned with their beliefs. The absence of culturally competent services led them to rely on their own health systems, which often restricted their perceived control and hindered effective help‐seeking [17].

Models of care delivery for chronic diseases to bridge gaps between the acute healthcare sector and community‐based services are fundamental to providing comprehensive and integrated care. Care pathways linked to primary care settings aim to connect care providers across the care continuum, addressing fragmentation issues [7, 13]. In addition, models for managing acute conditions and chronic diseases highlight the necessity for patients to play an active role in decision‐making regarding their health [18]. Effective treatment and enhanced chronic care services necessitate patient–provider partnerships within an integrated system of collaborative care. Such partnerships empower patients to engage with healthcare providers, enabling them to discuss their health issues and develop problem‐solving skills and self‐efficacy [18, 19, 20].

Incorporating the perspectives of patients and their families is crucial not only in the clinical interface but also in designing services that acknowledge underlying issues related to the real‐world challenges they encounter. This approach aims to achieve sustainable improvements in health‐system performance, aligned with people's needs, preferences and cultural contexts, all while working within the constraints of available healthcare resources [19, 20]. This is where health service design methodologies that are flexible, incorporating real‐world experience based on multiple inputs can be highly valuable in developing tailored, culturally appropriate interventions for disease management that align with patients' needs and healthcare resources [7, 13]. Codesign is a well‐established approach to designing solutions in which key stakeholders contribute equally to the design process, enabling people to communicate and cooperate across disciplines and between organisations [21, 22]. The World Health Organisation recognises the importance of patient empowerment, and patient‐centric and collaborative approaches to the redesign of health services [23]. However, in LMICs, application of these approaches has been limited [14]. Health service delivery models tend to have limited consumer involvement in the design and delivery processes [20]. Bridging the gap requires that care delivery models for CHF need to be context‐specific and provides opportunities for individuals with lived experience to participate in the service design process, thereby overcoming obstacles they face in accessing healthcare services [19, 20].

The codesign approach is well‐established in developed countries and regions such as the USA, Europe and Australia [24]. Although a small number of codesign studies have been successfully performed in developing countries, predominately in Nepal, Ethiopia and Egypt [25, 26, 27], cultural and hierarchical factors are significant potential barriers to equitable collaboration in service re‐design [28]. These factors need to be considered when attempting collaborative methodologies.

This codesign study was conducted in an acute care hospital in Bangkok, Thailand, where organisational leaders and clinicians were dealing with a high rate of CHF readmissions and recognised the limitations of the existing care delivery model that offered very little disease management support for people with CHF and no access to multidisciplinary CHF clinics [29], cardiac exercise rehabilitation [30] or community follow‐up programs [12]. The study aimed to codesign a tailored, evidence‐based CHF disease management support service that would be feasible, acceptable to all stakeholders (clinicians, organisational leaders and patients), and viable in a specific resource‐limited context. In this article, we report the processes and outcomes of the design thinking workshop component of this multi‐faceted study.

## 2 Methods

### 2.1 Setting

The codesign study was conducted in a 500‐bed tertiary hospital in Bangkok, Thailand. This health service provided care to people living in urban areas and received referrals from regional Thailand. Health service leaders had identified significant challenges in providing ongoing support for individuals living with CHF due to limited resources, resulting in high rates of acute care readmissions [4].

### 2.2 Study Design

**Figure 1:**
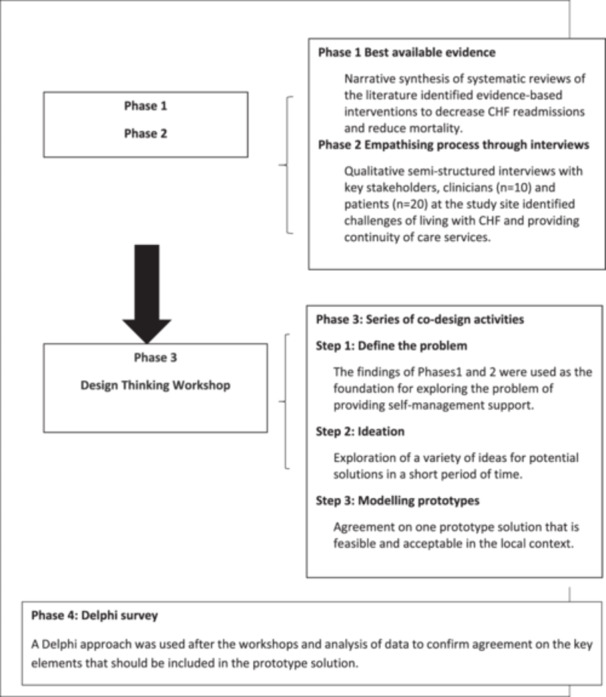
Outline of the four phases in codesign, including the three steps undertaken as part of the Design Thinking workshop.

The overall codesign approach of this study comprised four phases (Figure 1) occurring between February 2021 and June 2023: (1) identification of the evidence‐base through a narrative synthesis of systematic reviews of disease self‐management support (DSMS) programs and their effects on survival and readmissions [14], (2) qualitative, semi‐structured interviews with clinicians (*n* = 10) and patients with CHF (*n* = 20) in the study hospital to identify real‐world challenges, conducted between February and May 2021 [4], (3) codesign activities applying the principles of design thinking to develop a prototype solution to challenges faced, and (4) a follow‐up Delphi survey to refine and define the key elements of the proposed solution [31]. This article presents the methods and outcomes of Phase 3 of the codesign process, the design workshop.

The codesign workshop activities were conducted using a design thinking approach and user‐driven development process to cocreate a solution [32, 33]. The Design Thinking model proposed by the Hasso–Plattner Institute of Design at Stanford University has been used widely to redesign healthcare delivery [33]. The model aims to create meaningful innovations through a user‐centred design process involving users and care providers within the context of their challenges [33, 34]. A distinctive feature of the design thinking process is the emphasis placed on establishing empathy with patients and clinicians' experience of the problem [33]. This is the first stage of the process and is fundamental to the next stage of ‘defining the problem’. The stages that follow facilitate creative, divergent thinking to identify potential solutions suitable for implementation and testing in the real‐world setting (Stage 3, ideation) and then, in the ‘prototype’ stage, using critical thinking approaches, to select the most applicable and feasible solutions [32]. Activities are highly intentional whereby ideas are represented physically by sticky notes, storyboarding and visual matrices, thereby giving equal value to each participant's ideas [33, 35, 36]. In our study, we applied four of the five stages of the design thinking model to inform the codesign workshop (Table 1). The future, final stage will be to test the prototype.

**Table 1:** Design thinking steps and techniques used in codesign workshops.

Design thinking stages	Description	Workshop activities
**Empathise**	This stage explored the needs and challenges of stakeholders to inform problem identification through empathy. These insights were foundational to the workshop task of developing a tailored, feasible and sustainable solution through subsequent Codesign activities.	Activity 1: Pre‐workshop: Qualitative individual and focus group interviews with key clinical stakeholders and patients at the study site provided a deep understanding of end‐users’ needs and challenges that needed to be addressed and assisted in defining the problem. A visual, summary infographic was provided to participants (Figure S1).
**Define the problem**	This stage involved a synthesis of the challenges and needs identified through the empathy work and framing these challenges to reach agreement of the target needs to be addressed in a tailored model of care.	Narrative synthesis of the systematic review literature of the effectiveness of DSMS programs in reducing mortality and health service utilization provided evidence‐based information about potential service improvements. A visual, summary infographic was provided to participants (Figure S2).
		Workshop activity:
		Whole group open discussion, sharing information, experiences and perceptions of care to facilitate a shared understanding of the problem. The discussion was facilitated using point‐of‐view questions like, ‘*What is the patient experience*?’ [37]. Participants were encouraged to pool their ideas and insights by writing them on sticky notes that were then placed on a whiteboard to promote further discussion. 30 min were allocated to this activity.
		The outcome was a focused problem statement.
**Ideate and develop prototype solutions**	This stage is where innovative ideas or solutions are generated by leveraging different perspectives and strengths of participants. It is achieved through a variety of techniques including brainstorming to promote divergent thinking among participants. This process encouraged creative thinking and promoted a sense of collaboration.	Activity 2: Participants split into two self‐selected groups and were encouraged to generate potential solutions suitable for implementation and testing in the real‐world setting. Participants were asked to brainstorm and select the best solutions to advance to the prototyping stage. To facilitate the process, two matrices were used: **Creative matrix,** a 5 × 5 cell matrix with columns representing people involved in the potential solution represented in the categories in the rows) to help generate a wide range of concepts in each cell [37] (Table S1); Sticky notes facilitated this process; **Importance versus Cost** matrix (a 2 x 2 matrix). This matrix was used to reduce the number of potential solutions (Table S2)
**Modelling prototypes**	This step aimed to identify the best possible and feasible solution of those identified in the ideation stage. This process involved patient journey mapping to determine which solution best met the needs and challenges identified earlier in the process.	Activity 3: Participants worked with a partner to create a draft version of one possible solution selected from the importance/cost matrix. Storyboarding was used to map the proposed solution in terms of the patient journey [37] During feedback sessions, participants were asked to share their thoughts about the feasibility of their solution in terms of service requirements and personnel responsibilities. At the end of the feedback session, participants indicated their preferred solution by placing coloured dots on the matrix.

### 2.3 Ethical Considerations

Approval to conduct the study was obtained from the hospital review board in Bangkok, Thailand (LH631044) and the Human Research and Ethics committees of Deakin University (HEAG‐H 39‐2021). Participants provided voluntary and signed informed consent.

### 2.4 Participants and Recruitment

A purposive sample of HF clinicians, organisational leaders and patient consumers was the intended configuration of participants in the study. Eligible participants were as follows:

1. Recent past patients who participated in Phase 2 interviews within the previous year [4]. These patients were aged 18 years or over with a confirmed diagnosis of CHF for at least 6 months, able to communicate and write in Thai language, and willing to participate in a workshop.
2. Healthcare professionals who were clinicians working with patients with CHF directly or as leaders, identified by the organisation as holding decision‐making responsibilities in regard to the design and delivery of CHF care services.

Recruiting participants was challenging as the research team was based in Melbourne, Australia while the participants who contributed to the design thinking process were based in Thailand. However, the majority of eligible healthcare service participants and all eligible patients were known to the primary author (A.K.) as she had conducted the Phase 2 interviews in person. A peer researcher (a cardiac nurse) in Thailand coordinated recruitment of participants by liaising with the Director of Nursing to identify potential participants and sending out emails.

The 20 patients who had participated in the Phase 2 interviews were contacted by the research team representative via telephone and invited to attend the workshops. The recruitment processes for Phase 2 have been described previously [4].

One month before the workshop, organisational leaders and clinicians (*n* = 75) were sent a detailed email inviting them to express interest in participating in the design workshop. Potential participants were: the Deputy Director of Nursing (*n* = 1), nursing operations directors (*n* = 5), Head of Dietetics (*n* = 1), pharmacists (*n* = 2), physiotherapists (*n* = 2), cardiologists (*n* = 3), Nurse Unit Managers (*n* = 8) and clinical nurses in cardiac medical/surgical wards, emergency department and rehabilitation wards (*n* = 53). These included 10 clinicians who had participated in the Phase 2 interviews (a cardiologist, a cardiac nurse, a pharmacist, a dietitian, three ward nurses and three intensive care nurses).

One week before the scheduled codesign workshop, 10 healthcare professionals (a cardiologist, a cardiac nurse, a pharmacist, a dietitian, three ward nurses and three intensive care nurses) and three patients with CHF from Phase 2 had agreed to participate. Additionally, six clinicians and two organisational leaders confirmed their interest in participating. This represented 24% of the clinicians and organisational leaders invited. The three patients withdrew from the Codesign activities on the day before the workshop due to either increased severity of their illness, and/or readmission to hospital. The peer researcher then attempted to contact other eligible patients by approaching three cardiac inpatients awaiting hospital discharge, and one was willing to participate. The final workshop participants were 19 individuals consisting of two organisational leaders: the Deputy Director of Nursing and the Chief Executive Officer of Strategic Planning for Nursing. The 16 clinicians who attended were a cardiologist, a dietitian, a pharmacist, a cardiac nurse consultant, medical critical care nurses (*n* = 4), surgical critical care nurses (*n* = 2), home care nurses (*n* = 2) and medical general care nurses (*n* = 4). There was one patient participant (Table 2). All participants expressed a preference to attend the workshop in person.

**Table 2:** Characteristics of workshop participants (*n* = 21).

Characteristics	*N (%)*
**Age group (years)**	
≤ 30	1 (4.8)
31–40	9 (42.9)
41–50	7 (33.3)
51–60	4 (19.0)
**Sex**	
Male	2 (9.5)
Female	19 (90.5)
**Role/discipline**	
Organisational leaders	
Deputy Director of Nursing	1 (4.8)
Chef Executive Officer of Strategic Planning for Nursing	1 (4.8)
Clinicians	
Cardiologist	1 (4.8)
Dietitian	1 (4.8)
Pharmacist	1 (4.8)
Cardiac nurse consultant	1 (4.8)
Medical critical care nurse	4 (19.0)
Surgical critical care nurses	2 (9.5)
Home care nurses	2 (9.5)
Medical general care nurses	4 (19.0)
Individual with CHF	1 (4.8)
Peer researcher (observer)	1 (4.8)
Researcher (PhD candidate)	1 (4.8)
**Practice experience (years)**	
11–15	4 (19.0)
≥ 16	17 (81.0)

### 2.5 Study Procedures

A detailed summary of procedures related to the design thinking approach is presented in Table 1. One week before the design workshop, participants were sent two, one‐page summaries of the findings of Phases 1 and 2 of the codesign study presented as visual infographics. The findings of the semi‐structured interviews in Phase 2 were presented as themes and illustrative quotes. This infographic supported the ‘empathy’ and ‘problem identification’ components of the design thinking process (Figure S1). This was a particularly important component given the limited patient participation in the actual workshop. The second infographic was a one‐page summary of the narrative synthesis of the components of effective CHF DSMS programs including the effectiveness of low to high‐intensity interventions [14] (Figure S2). This information provided participants with valuable insights and evidence‐based guidance to support ‘ideation and prototype’ discussions.

The duration of the codesign workshop, conducted February 20, 2023, was 2.5 h on a single day. The duration of the workshop was a consideration in balancing wanting to enhance attendance of clinicians and organisational leaders with allowing for depth of discussion. Provision of the infographics in advance that addressed important elements of the discussion was a significant design feature to center discussion towards a solution. The focus of the workshop was to conduct activities related to ‘defining the problem’, ‘ideation of solutions’ and agreement on a ‘prototype’ (Table 1). Workshop activities were facilitated by the first author (A.K.), a Thai national and Registered Nurse who had undergone education in Codesign thinking facilitation: (1) Design Thinking for Innovation and (2) Facilitation of Human‐Centered Design Practitioner through the LUMA Institute. The peer researcher was an observer.

#### 2.5.1 Activity 1: Defining the Problem

The first activity aimed to identify issues, gain insights and reaffirm the challenges associated with providing ongoing care to patients with CHF. The infographics were used to support participants in their discussions of the limitations of current health services and how the service was perceived by all stakeholders. Whole group discussion was encouraged and the intent was to use questioning to narrowly focus the discussion to an actionable problem statement that synthesised the specific, meaningful challenges involved and to promote convergent thinking regarding the problem that needed to be solved.

#### 2.5.2 Activity 2: Ideation and the Development of Prototype Solutions

The second activity involved brainstorming and sharing ideas to generate a wide range of potential solutions to the agreed‐upon problem statement. Participants were separated into two self‐selected teams. Importantly, the researcher suggested that each team (two groups of 10 and 9) represent diverse disciplines. The researcher facilitated the development of a creative matrix (Table 1). This session promoted divergent thinking through brainstorming to generate multiple ideas from various inputs and discussion of the possible solutions that should be feasible in the relevant context. The researcher nominated a team member to summarise each team's key ideas and provide more details to the entire group about each proposed solution.

An iterative process of discussion reduced 96 possible overlapping prototypes generated by the two groups to 10 then nine through a process of refinement and integration, considering impact and cost‐effectiveness. This was achieved by plotting the 10 ideas onto a 2 × 2 matrix of importance/difficulty matrix [37] (Table S2). Perceived importance referred to participants' views of the potential for a solution to address the problem, and difficulty referred to feasibility in terms of personnel, technology and cost. This matrix tool enabled quick assessment of the value of ideas and, which ideas should be prioritised for execution and prototyping in the next session. At the end of the activity, during the group summary feedback session, most participants reflected on the potential of using digital technological communication such as ‘LINE’ application to facilitate ongoing monitoring of patients' stability. This activity lasted 60 min.

#### 2.5.3 Activity 3: Modelling Prototypes

The aim of the third activity was to attain agreement on one feasible prototype solution to test at the study site. In groups of two or three, participants selected one possible solution from the nine identified in the previous activity by taking a sticky note off the importance/cost matrix. ‘Patient journey mapping’ was intended to outline the key elements of a care service and describe the individuals who would take responsibility for developing and implementing each step and those who would be directly affected (as either providers or recipients of the care).

Participants were then invited to present their storyboards to the group and all participants had the opportunity to discuss and evaluate the relative merits and feasibility of the solutions proposed through the storyboarding. Once all storyboards were discussed, participants voted for one solution to be developed and tested at the study site.

The intent of all the activities was to facilitate rapid design and evaluation of ideas to identify an ‘optimal’ solution to the agreed‐upon problem. Before the quick poll using anonymous sticky notes as voting tokens, participants were instructed to vote based on the viability of each prototype. This democratised the decision‐making process [37]. Visually representing the vote ensured that all voices were heard and considered, fostering a sense of inclusivity and ownership in the decision‐making process among the participants. This activity lasted 60 min.

### 2.6 Data Collection

The researcher (A.K.) facilitated the codesign activities and directed participants. The workshops were conducted in Thai language. Data were collected in collaboration with workshop participants. With participants' permission, the workshop's whole group discussions and feedback sessions were video and audio recorded for later analysis and all sticky notes and storyboards were photographed and collected. The peer researcher circulated between groups recorded field notes and collected physical data from whiteboards etc and assisted with the audio recordings of whole group feedback and discussions.

Audio recordings, field notes and sticky notes were transcribed and checked for accuracy by the interviewer (A.K.) and then translated into English language for subsequent analysis.

### 2.7 Data Analysis

**Figure 2:**
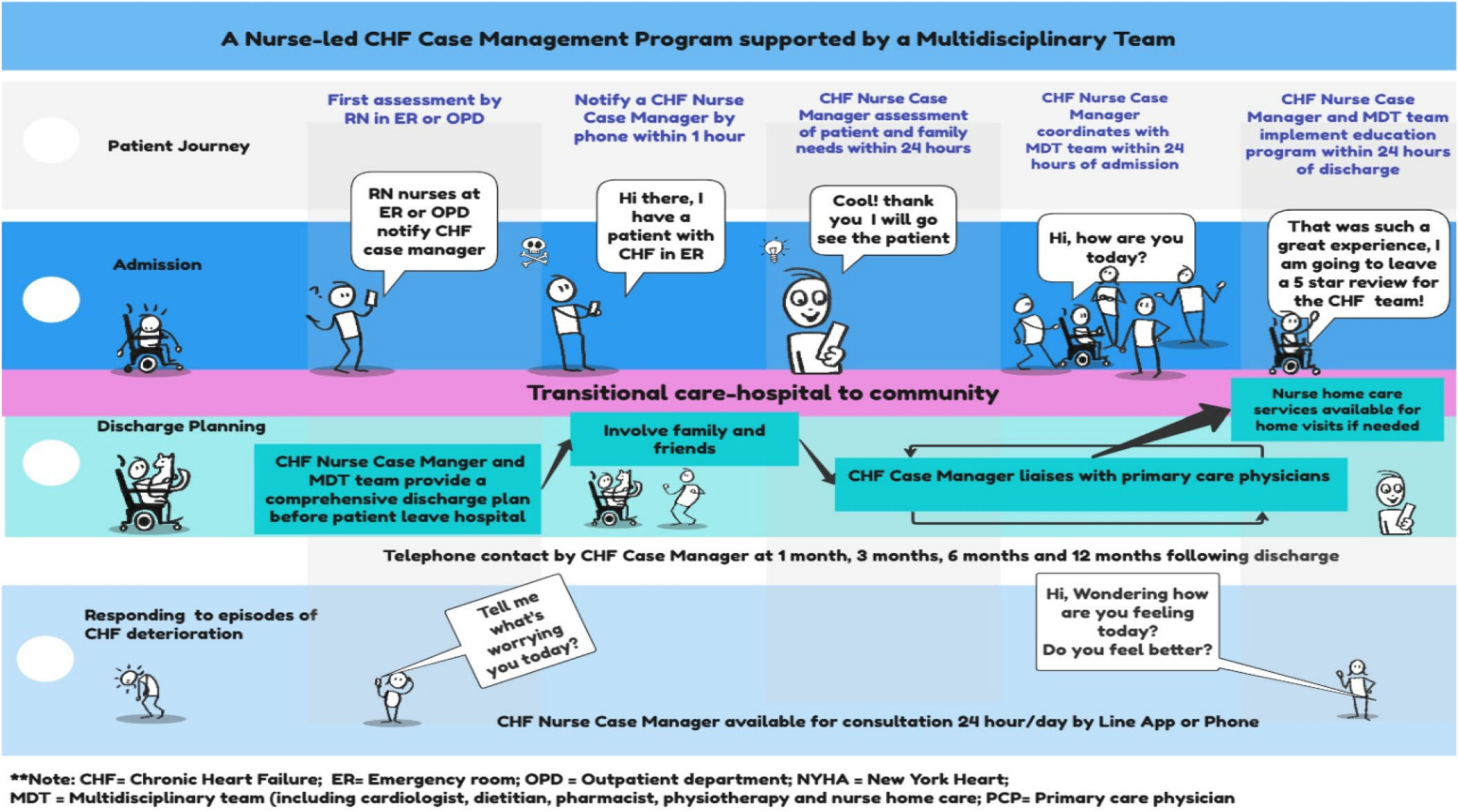
The proposed nurse‐led CHF case management program supported by a multidisciplinary team.

The purpose of the postworkshop analyses was to synthesise and summarise the processes and outcomes of the workshop to progress to Phase 4 of the program [31]. The outcome of the workshop was the agreed prototype solution, and this is illustrated in Figure 2. Analysis of the transcripts of group discussions provided a process record of workshop deliberations as well a summary framework of the key ‘themes’ discussed. Audio recordings were transcribed and checked for accuracy by A.K. These transcriptions were translated from Thai into English. The accuracy and authenticity of both the Thai and English versions were verified by a language expert based in Thailand. The English translation was refined until the language expert confirmed it accurately reflected the original Thai version.

Audio and physical data were analysed using content analysis, based on the process summarised by Kiger and Varpio [38]. The initial step involved reading the transcripts multiple times to enable familiarisation with the content. Next, a coding frame was developed based on the problem statement emerging from Activity 1. The problem statement had three core elements: continuity of care, access to and consultation with a multidisciplinary team and community outreach. Initial codes were generated by making margin notes and labelling data according to the coding frame. In the third step, the codes were organized according to the coding frame and themes were generated by identifying unifying patterns. The fourth step entailed reviewing the data to identify any emerging themes not coded against the coding frame and naming and defining themes identified. A.K. performed the initial sorting and coding of data. Academic research team members (M.B. and A.H.) reviewed the transcripts, engaged in discussion about codes and themes and verified representative transcribed quotations in relation to the identified themes. Agreement on the final themes was reached through discussion and review of the data. The raw data and coding documents provide evidence of the analytical process.

## 3 Findings

The characteristics of the 19 participants in the codesign activities are described in Table 2. All participants stayed for the duration of the workshop. These were: two organisational leaders, 16 clinicians, one individual with CHF and two members of the research team (A.K. and the peer researcher, a cardiac nurse).

### 3.1 Definition of the Problem

Participants acknowledged and confirmed the findings from the Phase 2 qualitative study. They identified the current lack of ongoing support for people with CHF to self‐manage their disease, and the need to address the gaps in chronic care services, particularly in relation to continuity of care, multidisciplinary communication and collaboration and community outreach.

### 3.2 Potential Solutions and Selected Prototype

Participants were in agreement that the solution needed to be an integrated system for the coordination of acute healthcare and community care services for patients hospitalised with CHF that would improve the quality of chronic care services and patients' experience of care and provide patients with opportunities for timely treatment of exacerbations of their disease to prevent unnecessary hospitalisations.

During Activity 3, nine storyboards were developed outlining potential prototype solutions. Some storyboards had significant overlap: (1) a nurse‐led CHF case management with support from a multidisciplinary team (MDT), (2) discharge planning involving a community services program (*n* = 2), (3) an effective discharge plan followed by a home visit, (4) a planned discharge program with caregiver education, (5) ongoing telehealth support for patients with CHF program, (6) a family home healthcare, (7) a one‐stop CHF clinic service and (8) a CHF clinic and home visits (Table S3).

Voting to select the prototype solution to implement and test resulted in strong endorsement for two solutions: (1) a nurse‐led CHF case‐management service (*n* = 6) and (2) a multidisciplinary outpatient one‐stop CHF clinic (*n* = 6). Discussion followed that included the organisational leaders, who represented the financial decision‐makers in the hospital. The two organisational leaders negotiated a compromise with participants that the prototype solution should account for limited personnel, funding, resources and hospital space. Participants agreed to proceed with a nurse‐led CHF case management service supported by an MDT (Figure 2). Participants discussed how this service would improve care coordination, patient education and long‐term follow‐up in the community. The goal of the service was to empower patients to engage with healthcare providers and develop confidence to manage their condition and maintain disease stability in the community.

Analyses of the workshop transcripts of discussions and feedback sessions revealed five themes in relation to target activities going forward and also provided validation of the final prototype solution and the substantive evidence derived through the pre‐workshop phases of the codesign process. These themes were (1) need effective strategies to improve communication and provide greater access to multidisciplinary expertise; (2) need to engage multidisciplinary teams for proactive health delivery; (3) need to provide patients with CHF education about evidence‐based lifestyle changes and self‐management strategies; (4) need services that provide ongoing care to monitor and maintain illness stability and support patients during instability; (5) need continuity of care and greater engagement with the community and primary care providers. These themes are presented in Table 3.

**Table 3:** Summary of the key themes and targeted interventions emerging from workshop discussion.

Problem identification and target interventions			Ideation and prototype solutions		Prototype components
Themes	Defined challenges	Illustrative quotes	Possible solutions	Illustrative quotes	
Need effective strategies to improve communication and provide greater access to multidisciplinary expertise	There are challenges to working collaboratively as a multidisciplinary team due to inefficient communication and lack of leadership.	*We work solo and we only communicate with each other when we need consultation from others. It is difficult to work collaboratively because we do not have a leader for our HF care, and we do not have a HF team yet. (ID16, aged 44 years, 19 years of experience in CHF care)*	Instigate processes and strategies to improve communication between the MDT	*We (healthcare professionals) may need to improve our communication because we have little communication and consultation. (ID17, aged 36 years, 10 years of experience in CHF care)*	CHF Nurse Case Manager coordinates the multidisciplinary Regular MDT team meetings. MDT review of patients who have frequent readmissions.
		*We do not know who wants the responsibility of leading the CHF multidisciplinary team, as it seems like no one wants to take a leading role. Communication seems to be top‐down, and we have been waiting for the head to say who will take responsibility for leading and establishing a CHF team*. (ID07, aged 42 years, 15 years of experience in CHF care)			
**Need to engage multidisciplinary teams for proactive healthcare delivery**	Multidisciplinary involvement is needed and outreach is required to respond to the needs of patients with CHF.	*We do have expertise in specific fields, and we work separately. It is difficult to bring everyone together or spend time for commitment consensus to establish a multidisciplinary CHF hospital‐based team*. (ID16, aged 44 years, 15 years of experience in CHF care) *There is a lack of an interdisciplinary approach in which team members from various disciplines collaborate to address the holistic care needs of CHF patients and families.* (ID14, aged 52 years, 29 years of experience in CHF care) *As a tertiary hospital in the heart of Bangkok, we need to establish a CHF team. We need to form a team and decide who will be in it and their responsibilities.* (ID05, aged 44 years, 20 years of experience in CHF care)	Multidisciplinary involvement in outreach. Development of holistic treatment plans that address patients’ diverse needs.	*I am [an ICU nurse] eager to join the team, and everyone can create and change it, and we can proceed to the next stage of improving the quality of CHF services.* (ID05, aged 44 years, 20 years of experience in CHF care) *A CHF clinic will provide equal opportunity to work; we require a CHF team to improve our patients’ experiences with CHF services.* (ID16, aged 44 years, 20 years of experience in CHF care)	Nominate members of the MDT team at the hospital. Establish a multidisciplinary CHF clinic. CHF Nurse Case Manager liaises with home care nurses and primary care providers.
**Need to provide patients with CHF education about evidence‐based lifestyle changes and self‐management strategies**	It is challenging to transfer evidence‐based and inconsistent education in a setting with patients from diverse cultural backgrounds and poor referral practices.	*I am [dietitian] willing to provide specific information about dietary recommendations before discharge and follow‐up. It will be easier if we have a pathway of care and alerts to CHF case admissions.* *(ID17, aged 36 years, 10 years of experience in CHF care)* *I lacked self‐management and HF education. For example, last year, my legs were swollen several times, and I had never weighed myself. But I realised something was wrong, and I decided to take diuretics straightaway. So, I was rehospitalised four times last year because sometimes the diuretics were not helpful. Patients with HF really do need support when seeking help or consultation.* (ID19, aged 24 years, living with CHF for 5 years)	Healthcare providers and services need to use multidisciplinary knowledge to address the challenges of supporting self‐management for patients from diverse backgrounds	*As every patient is different due to their needs, cultural beliefs and level of income, we need to develop a program that includes patients, their families and community facilities to ensure that the education provided meets their expectations and support networks may help us reinforce education and encourage patients to perform proper self‐care as individuals.* (ID04, aged 49 years, 26 years of experience in CHF care) *Establish a CHF team to provide education and allow these patients to access our future CHF team, which should be available 24/7 for response if needed after the patient is discharged.* (ID02, aged 60 years, 30 years of experience in CHF care)	Develop a tailored, culturally appropriate CHF educational program based on individuals’ culture, needs and preferences to ensure that the information is suitable for patients from diverse backgrounds with variable health literacy skills. Established a multidisciplinary CHF clinic.
**Need services that provide ongoing care to monitor and maintain illness stability and assist during instability**	Sharing information with patients and healthcare providers across the care continuum to coordinate care, provide a holistic approach and, enable ongoing patient monitoring to reduce deterioration and the need for readmission.	*Previously, I dealt with difficulties in access [care] after discharge and suffered because I did not know whom to contact even though I work at the hospital. Patients need help to form a connection between the hospital and their homes.* (ID19, aged 24 years, living with CHF for 5 years)	Use digital technology to enable the provision of ongoing supportive care in the community	‘*Technology may assist the team in establishing holistic care approaches by making healthcare systems more accessible.’* (ID14, aged 52 years, 29 years of experience in CHF care) *Technology has become essential to provide patients and families with a holistic care approach. Innovative technology, such as Telehealth, may allow patients and their families to access their healthcare systems after they are discharged*. (ID17, aged 36 years, 13 years of experience in CHF care)	Providing patients with access to E‐Health CHF education website. Establish the LINE application for daily monitoring and maintain disease stability. Developing communications technology for long‐term follow‐up.
**Need continuity of care and greater engagement with the community and primary care providers**	Establishing an ongoing partnership care plan requires collaboration with community services and local leaders.	*We do not know how they are after being discharged home as we do not link with their local community services in every case.* (ID06, aged 38 years, 13 years of experience in CHF care)	Engage local community leaders and primary care providers to promote community engagement and improve continuity of care	*To build an ongoing partnership care plan, we need to work with community services or a local community leader*. (ID03, aged 49 years, 20 years of experience in CHF care).	Case management Nurse coordinates the home care nurses. Case management Nurse liaises with primary care providers.
					

## 4 Discussion

To our knowledge, this is the first codesign study to systematically approach redesign of a health service for patients with CHF within an acute care hospital in Thailand. The codesign workshop was successful; participants contributed actively during activities, fostering a sense of teamwork and ownership of the solution. At the conclusion of the codesign activities, an agreement was reached to develop a nurse‐led, CHF self‐management support service supported by an MDT that included coordinated care, patient education and ongoing disease‐management support in the community.

Codesign encompasses meaningful engagement of multiple stakeholders with diverse perspectives sharing information and fosters discussion and collaboration. This collaborative approach has been shown to improve the integration of care and health service outcomes in resource‐limited settings [20] although the evidence is limited. Achieving positive care outcomes involves collaborative design that facilitates user discussions, negotiations and decisions close to the point of care. This approach aims to develop tailored service delivery models that align with specific local needs and resources and as such are more likely to be successful [19, 20, 24]. In this codesign study, participants had their first opportunity to work collaboratively as one team to redesign a health service they acknowledged was in need of reform. The structured and systematic codesign and design thinking approach leveraged real‐world experiences from various sources to develop a tailored disease management prototype program that aligned with patients' requirements and the healthcare system's capabilities. If implemented the proposed model should enhance healthcare delivery even with limited resources. Inclusion of organisational leaders ensures that the redesign aligns with available resources, and incorporating best available evidence assists in tailoring interventions to improve their cost‐effectiveness [19, 20]. In this study, there was substantive agreement between patients, clinicians and organisational leaders about the challenges and limitations of existing services, and the target interventions required to achieve integrated care for people with CHF [20]. The proposed solution focused on discharge planning and care coordination, the establishment of a multidisciplinary team and the development of a chronic care model placing patients at the centre of service design and care delivery.

The codesign process of providing a synthesis of the evidence underpinning disease self‐management support programs, albeit mostly derived from high‐income countries, and themes derived from interviews with key stakeholders meant that participants were assisted through a structured, fully informed process. This triangulation of data was an efficient way to guide participants when generating multiple ideas for possible prototypes [20, 39]. The findings from Phase 1 highlighted the urgent need for robust, tailored, culturally acceptable disease self‐management programs for CHF in SE‐Asia [14]. Phase 2 data identified the needs and challenges of people living with CHF and the perceptions of health professionals involved in their care within a health service, providing compelling evidence for the importance of improving treatment optimisation, service delivery and access to community support services [4]. Codesign is a valuable process enabling healthcare providers and consumers to be creative contributors to the redesign process without requiring formal design training [40]. Storyboarding offers concrete and practical depictions of patients' journeys within a healthcare service, enabling the assessment of service quality based on evidence‐based principles and real‐life scenarios [41, 42].

The themes that emerged from the analyses of the group discussion and feedback sessions highlighted the need for coordination and continuity of care and communication between interdisciplinary team members. The absence of such coordination has led to incomplete and inconsistent patient education resulting in disjointed care and poor health outcomes for patients. Participants recognised the urgent need to address these limitations in the care provided through the coordination of care coordination and multidisciplinary support integrated with primary care services. Consistent with previous studies, LMICs must improve access to healthcare services and this requires engaging with healthcare leaders and managers [19, 43, 44].

Nurse case management is widely practiced in high‐income countries, proving effective in reducing readmission and mortality rates [14, 45]. However, directly applying nurse case management programs from high‐income countries to low‐ and middle‐income countries may not be feasible [14]. Effective nurse case management models in chronic illness require access to resources such as HF clinics, MDT and community care. Limited access could hinder achievement of health and health service utilization outcomes [46]. The codesign process offered the opportunity to shape the care coordination model to the Thai context and the local context specifically. Nurse case managers play a fundamental role in managing healthcare resources and improving communication between patients, families and healthcare providers [45, 47, 48]. Key aspects of nurse case management include predischarge planning to promote patient self‐management and support lifestyle changes through education and medication optimisation [45, 47, 48]. In addition, there is a focus on supportive post‐discharge follow‐up, early contact with patients post‐discharge and regular follow‐up for early detection of signs of deterioration [14, 47, 48]. Empowering nurse case managers to coordinate care fosters collaboration across interdisciplinary and primary care teams, addressing the need for improved leadership and greater access to multidisciplinary and continuous care support. This multidisciplinary approach also potentially enhances the delivery of culturally sensitive healthcare services that consider local climatic conditions, enhancing patient experiences and improving health outcomes.

While the specific elements of the CHF nurse coordination model will be determined in Phase 4 of the codesign process, the narrative synthesis conducted in Phase 1 identified the importance of the intensity of DSMS interventions in terms of their effect on healthcare readmissions and mortality [14] and was used as foundational guidance for shaping proposed solutions [20, 24]. This facilitated discussion regarding elements of high‐intensity interventions such as support and strategies to monitor HF symptoms and prevent serious exacerbations of the disease, and provision of 24‐h support for patients experiencing worsening of HF symptoms. Ideas relating to implementation included real‐time remote monitoring using LINE apps to improve patients' daily self‐management for early detection of deterioration and maintaining disease stability.

Through the codesign activities, participants identified and agreed on the challenges associated with providing care within the current service, the target interventions needed to address these challenges and a feasible model of care given constraints associated with limited resources, thereby enhancing the likelihood of successfully implementing the solution [24]. Engaging consumers in collaboration with healthcare services empowers them to cocreate new care models, thereby driving meaningful improvements in healthcare quality and consumer outcomes [25, 26, 27]. Involving consumers in the development and delivery of redesigned health services tailored to the needs of individuals living with congestive heart failure (CHF) enhances cultural acceptability [20, 49]. This codesign approach can be adapted to culturally diverse settings, addressing care challenges among various consumer groups and potentially reducing inequalities in accessing healthcare services [20, 49]. Nurse case managers play a crucial role in delivering personalized care tailored to the individual needs and self‐management capabilities of CHF patients, thereby addressing barriers to healthcare access. Importantly, an iterative testing process is essential to ensure the delivery of accessible care that meets local needs effectively.

### 4.1 Strengths and Limitations

This project used a design thinking approach to redesign a healthcare service, culminating in a nurse‐led CHF prototype with multidisciplinary team services aimed at supporting lifestyle changes across the care continuum. Conducted at a tertiary hospital in an urban area, the project engaged participants who provided valuable insights into real‐world challenges and facilitated the development of culturally acceptable and resource appropriate interventions aligned with their preferences in a short timeframe. The project successfully fostered collaboration among healthcare professionals in a dynamic design team dedicated to improving healthcare outcomes for an underserved population.

However, the study faced several challenges associated with codesign methods. First, being conducted at a single healthcare site in Bangkok, Thailand, limits the generalizability of findings to other CHF service users. The outcomes of this project may be particularly beneficial to Southeast Asian societies characterized by strong hierarchical and cultural beliefs as they undertake codesign initiatives to reshape healthcare services and apply knowledge into practice. Second, logistical constraints due to the research team's location in Australia and the planning challenges post‐COVID‐19 pandemic hindered participant recruitment for codesign activities, especially patients and their families at the hospital. Moreover, the lack of coordination between hospital and primary care services precluded the inclusion of the perspectives of a primary care setting in this study. Future research should engage the community and primary care sectors to gain broader perspectives and address emerging issues, potentially yielding better outcomes in restructuring models of chronic care services.

Thirdly, the lack of patient participation at the actual workshop was an important limitation. We sought to engage patients with HF who had high rates of readmission so that they could bring their experiences to the codesign activities. This proved a barrier to participation, as many were unable to attend due to exacerbation of their illness and/or admission to hospital. Future studies should ensure that the number of patient participants is proportional to other knowledge user groups.

Finally, while a proposal for the final solution was developed within the project timeline, funding constraints prevented the evaluation and testing stages of implementation. Future plans include implementing the nurse‐led management program and evaluating its effectiveness, feasibility and applicability in subsequent projects. Evaluating the codesign process is essential to ascertain whether it achieved the goal of enhancing healthcare in collaboration with key stakeholders. Assessing program effectiveness through pilot testing, iterative interviews with stakeholders, and measuring health outcomes such as literacy, knowledge and self‐care behaviours will provide insights into care delivery processes, sustainability and barriers to accessing healthcare services in the local context.

### 4.2 Recommendations

Globally, the main challenges for healthcare providers in low‐middle income countries, in managing CHF area access to robust, applicable practice guidelines, moving from an episodic acute care model to an integrated chronic care approach and working out how to apply international best evidence in their local practice setting [7]. Healthcare providers and policymakers need to consider the experiences of patients and their families when designing chronic disease management models for provision of ongoing long‐term supportive care in the community [7, 12]. Tailored chronic care interventions are necessary where there are cultural, resource and environmental challenges to be considered [50, 51]. Use of a codesign process can promote a user‐centred approach to chronic disease management by ensuring effective communication between clinicians and patients, thereby allowing consumers to share values, needs and preferences. Future research is needed to transform the tailored solution into implementation trials in real‐world settings to test the feasibility and acceptability of the intervention and whether it positively affects patient outcomes [52].

## 5 Conclusion

Using a codesign approach for the development of a chronic disease management service in Thailand, key stakeholders identified the importance of moving from a short‐term model of care to an integrated, multidisciplinary approach to providing long‐term support in the community. The final agreed prototype for health service redesign was informed by the best available evidence and the experience of patients and healthcare providers. The CHF Nurse Case Management service supported by a multidisciplinary team with a focus on community outreach addressed the key concerns of participants and was considered a feasible approach to developing a CHF chronic care service for the community in urban Bangkok, Thailand.

## Author Contributions

**Apinya Koontalay:** conceptualization, methodology, investigation, formal analysis, writing–original draft, data curation. **Mari Botti:** conceptualization, methodology, formal analysis, supervision, writing–review and editing, data curation. **Anatasia Hutchinson:** conceptualization, methodology, formal analysis, supervision, writing–review and editing, data curation.

## Conflicts of Interest

The authors declare no conflicts of interest.

## Supporting information

Supporting information.

## Data Availability

The data that support the findings of this study are available from the corresponding author upon reasonable request.
