# Supplementary material for: Development of a User‐Centred Chronic Care Model for Patients With Heart Failure in a Limited‐Resource Setting: A Codesign Study
Source: Health Expect. 2025 Jan 5;28(1):e70142. doi: 10.1111/hex.70142 (PMC11702422; doi:10.1111/hex.70142)
Supplement: Supplementary file 1 — Supporting information. [file HEX-28-e70142-s001.docx]

**Supplementary Figure 1** Infographic of the analyses of the semi-structured interviews conducted with patients, clinicians and organisational leaders


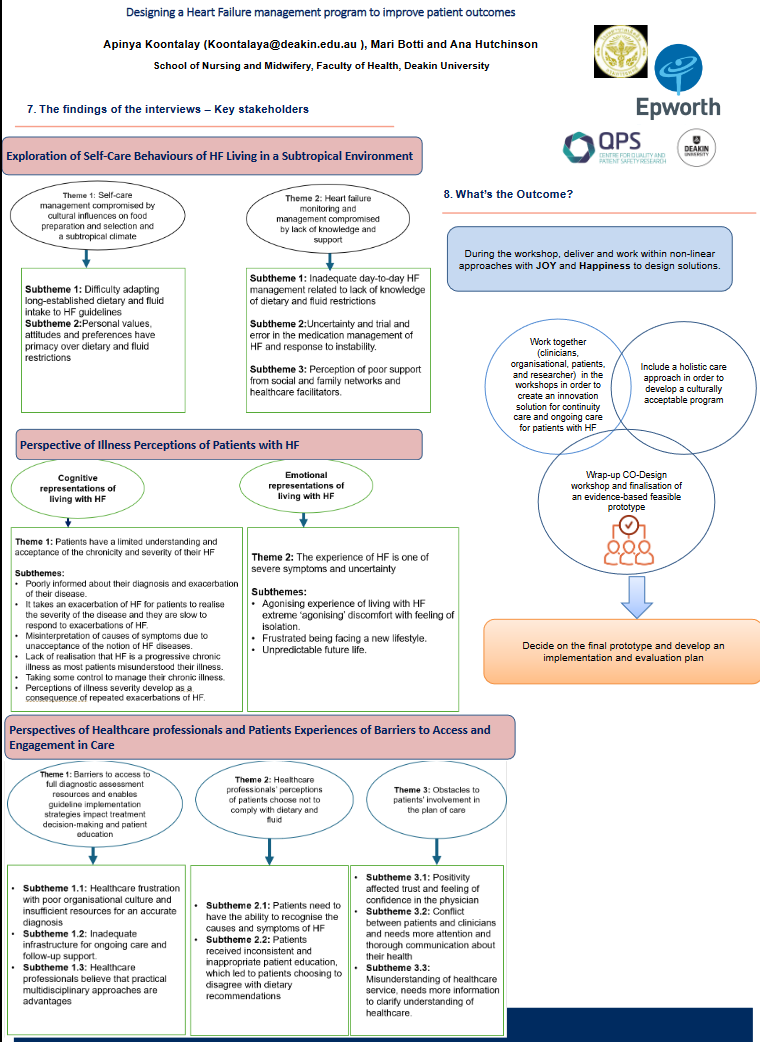


**Supplementary Figure 2** Infographic was a one-page summary of the narrative synthesis of the components of effective CHF DSMS programs including the effectiveness of low to high intensity interventions


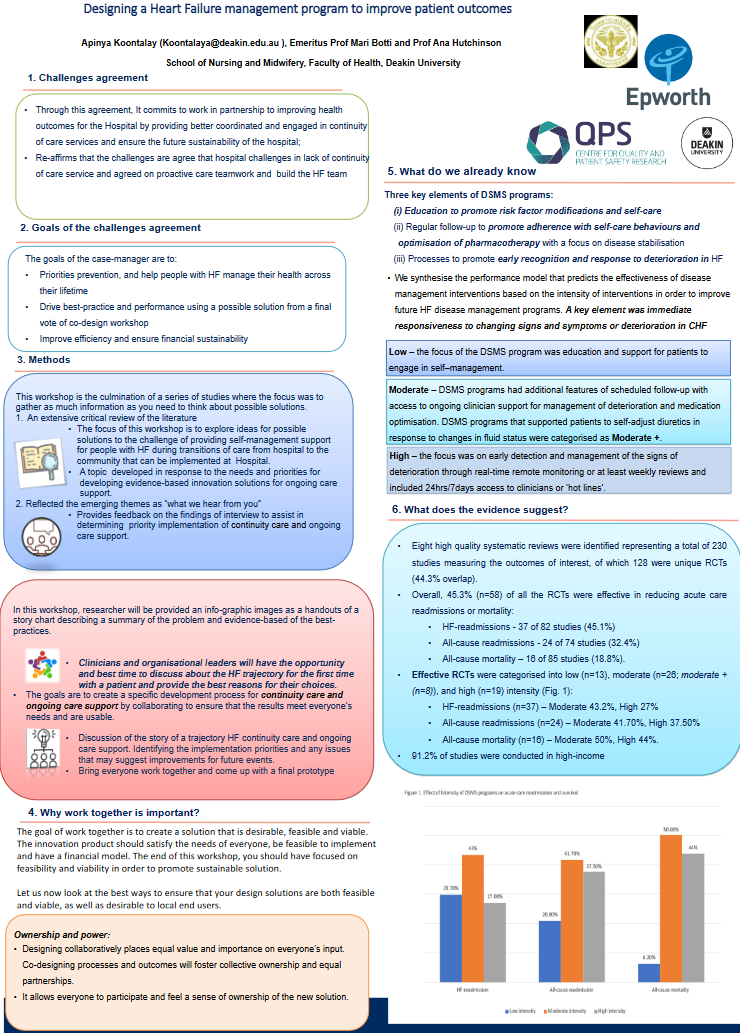


**Supplementary Table 1** **Activity templates for creative matrix**

| **_Guide_** |  | **_How might we never again leave a patient wondering what’s going on?_** | **_How might we eliminate reasons to complain about hospital food?_** | **_How might we enable patients to feel like an important part of their own care team?_** | **_How might we transform so that patients feel as cared for as a much loved family member?_** |
| --- | --- | --- | --- | --- | --- |
| **_Designate columns:_** _identify categories related to people_ | **_TECHNOLOGY&DIGITAL MEDIA_**   - _MOBILE DEVICE & WEARABLE TECH_ - _SOCIAL MEDIA_ - _INTERNET OF THINGS_ |  |  |  |  |
| **_Designate rows:_** _identify categories for enabling solutions_ | **_FACILITIES & ENVIRONMENTS_**   - _PERMANENT STRUCTURES_ - _TEMPORARY INSTALLATIONS_ - _VIRTUAL WORLDS_ - _MOBLIE ENVIRINMENTS_ |  |  |  |  |
| **_Ideate:_** _Ask participants to ideate at the intersections of grid_ | **_LIFESTYLE TRACKING_**   - _NUTRITION INTAKE_ - _PHYSICAL ACTIVITY_ - _SLEEP QUALITY_ - _THE QUANTIFIED SELF MOVEMENT_ |  |  |  |  |
| **_Helpful hints:_**   - _One idea per sticky note_ - _Limit the time to approximately 15 minutes_ | **_INTERNAL POLICIES & PROCEDURES_**   - _DIAGNOTICS & ASSESSMENTS_ - _INCENTIVES & REWARDS_ - _TRAINING & EDUCATION PROGRAMS_ - _COMPANY GUIDELINES_ |  |  |  |  |
|  | **_WILDCARD_**   - **_OUT OF BOX_** - **_ANYTHING GOES_** - **_+++_** |  |  |  |  |

**Supplementary Table 2** **Importance/Difficulty Matrix**

**
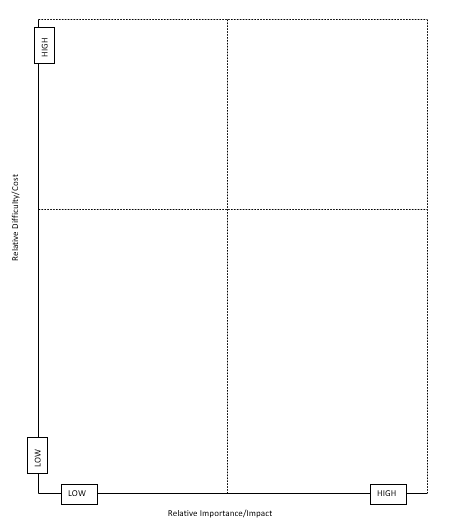
**

**Supplementary Table 3** Summary of proposed Heart Failure disease management models of care

| **Prototype model** | **Delivery stages of model of care** | **Recommended changes** | **Intervention plan** | **Team discussion** |
| --- | --- | --- | --- | --- |
| a nurse-led CHF case management with the support from a MDT team | Establishing case management pathway.  Support self-management and healthcare system to provide proactive care and follow-up | Suggestions for mechanisms to improve response and recognition to patient deterioration 24/7 hr/day | **In-hospital**   1. Ward nurses coordinate with CHF case manager within 1 hour of admission. 2. First visit on admission day within 24 hours. 3. Case manager coordinates with the multidisciplinary CHF team 4. Comprehensive discharge planning within 24 hours before discharge by a multidisciplinary CHF team for patients and caregiver 5. Case manager coordinating with the home healthcare for planning of a home care program before discharge.   **Outpatient remote monitoring provide by case manager:**   1. Coordinate with primary care services in patients’ areas. 2. Promote self-monitor and daily transmission of physiological data (weight, BP, HR). 3. Provides the consults via Line applications and available 24/7 hr/day. 4. Provides telephone support for reinforcement education at 1, 3,6 and 12 months.   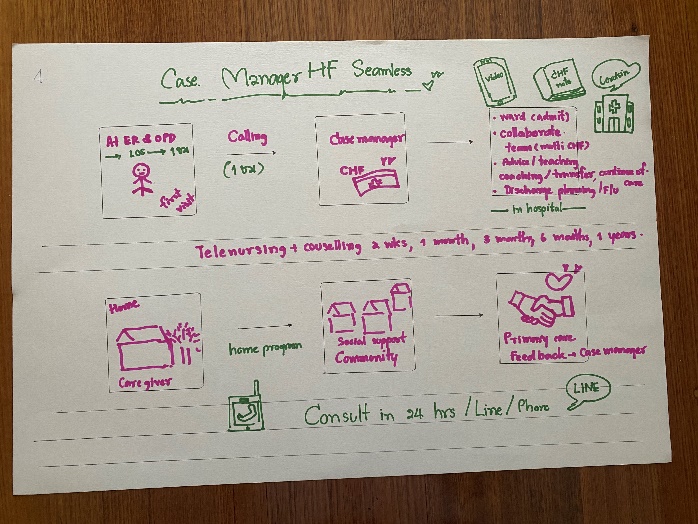 | 1. Teams proposed that this strategy was realistic and appropriate. 2. Technological innovations in healthcare to facilitate communication with patients and amongst the MDT. 3. This method may evolve to include a specialist CHF-clinic for specific subgroups. |
| Discharge Planning with involving community services Program | Establishing referral pathway to community resources. | Involvement of locally community base services | **In-hospital**   1. Consult a cardiologist before creating the first home visit in high-risk cases. 2. Provides the contact details for patients and family.   **Discharge and outpatient support program**   1. Nurse home healthcare provides the first contact with patients within two weeks by phone calls. 2. Nurse home healthcare linked with the local community services.   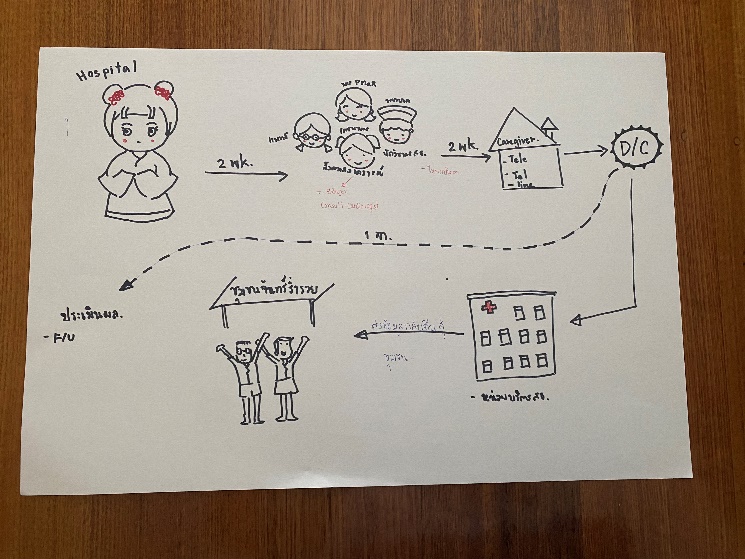 | The team proposed that a comprehensive discharge planning and assessment of individual requirements be incorporated to establish an individual care plan. |
| Discharge planning with involving community services program | Support self-management and education prior discharge to home, particularly in dietary advice | Proactive community care services and facilitator support for patients and families. | **In-hospital**   1. Predischarge assessment by a multidisciplinary CHF team and provision of a patient diary record for medication review, record physiological data and information about CHF 2. Consultation with dietitians before discharge.   **Discharge and outpatient support program**   1. Follow-up by telephone within 2 weeks 2. Motivate patients to self-monitor and review by CHF case manager. 3. CHF case manager consultation with multidisciplinary team if needs for treatment changes 4. Home visits at 1 month then every 3 months.   **Linked to community resources.**   1. Training village health volunteer for proactive care services such as provide daily weight or/and consulting with a multidisciplinary CHF team at the hospital if needed.   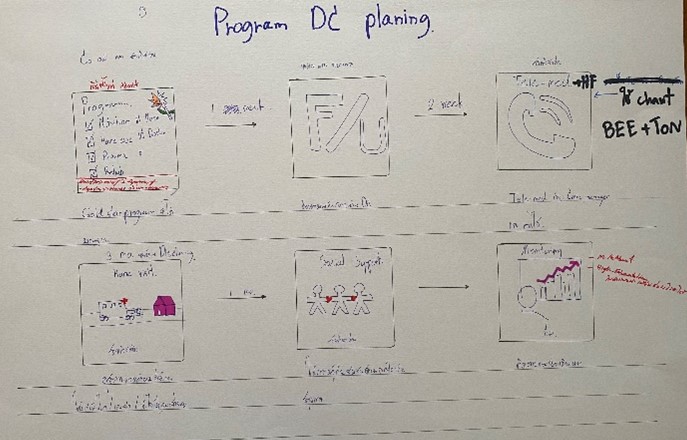 | 1. High-cost intervention and requires a significant number of healthcare providers. 2. Healthcare providers may face challenges of coordinated care by bringing multiple providers involved in the discharge care plan. |
| An effective discharge plan followed by a home visit | Integrate healthcare system and perform team for driven continuity of care for patients and family | Support education for patients and caregiver to gain their understanding about self-management skills by involvement families and community resources | **In-hospital provide by ward nurse**   1. Provide in-person education and written materials for patients and caregiver during admission. In particular: food preferences in order to promote healthy eating habits changes. 2. Develop the discharge planning application and multidisciplinary CHF team can access and provides treatment changes: (i) create individual care plan and discharge planning within 24 hours, (ii) provide education for patients and family while admission. 3. Comprehensive pre-discharge by a multidisciplinary CHF team.   **Discharge and outpatient support program by case manager**   1. Provide first contact by phone within 48 hours. 2. Available phone access during office hours (8 am to 5 pm, Mon to Fri) 3. A CHF clinic visit every 2 weeks or additional visit if required.   **Linked to community-based service for low risk case**   1. Home healthcare contact the community-based services in patients’ area to provide home visit if needs.   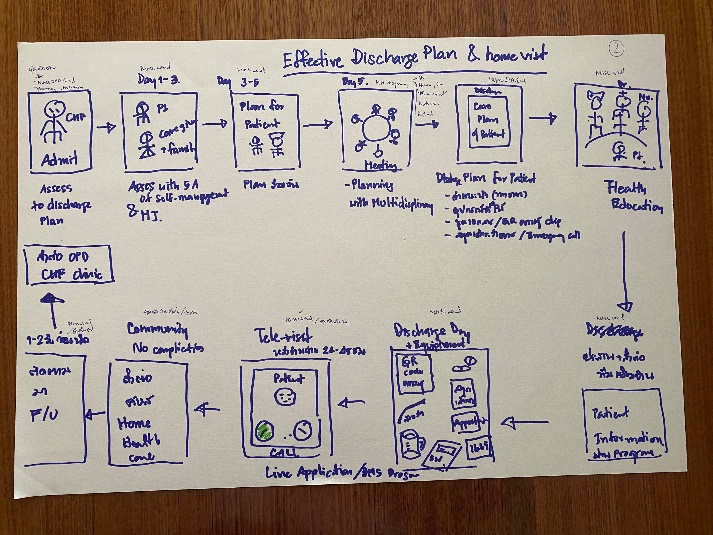 | 1. It appears unlikely that this program can be developed because it requires several staff members to contribute to the program. 2. Difficult to provide care through technology and to avoid wasting time. 3. Over workload which may affect patients’ safety and difficult to promotes continuity of care. 4. Costly |
| A planned discharge program with caregiver education | Establishing a multidisciplinary CHF pathway for monitoring and maintaining of disease stability | Healthcare costs benefits and patient’s and families’ satisfactions | **In-hospital**   1. Nurses ward provide education and materials for patients and caregiver during admission:   1.1 Day 1: assessment of caregiver knowledge and their understanding of cause and disease  1.2 Day 2: provide self-care education and CHF information by using teach back method.  1.3 Day 3: provide comprehensive discharge planning and materials education such as VDO  Provide healthcare contact details for patients and caregiver if needed.  **Remote monitoring provided by multidisciplinary team:**   1. Tele-nursing within 48 hours of discharge. 2. Telemonitoring through videoconference for monitor and maintain disease stability and nurse consult a multidisciplinary team if needed. 3. Promote self-monitor and daily transmits of the physiological data and review by case manager. 4. Pharmacists provide education and medication reviews within 2 weeks. 5. Provide medicine delivery and promote medication adherence.   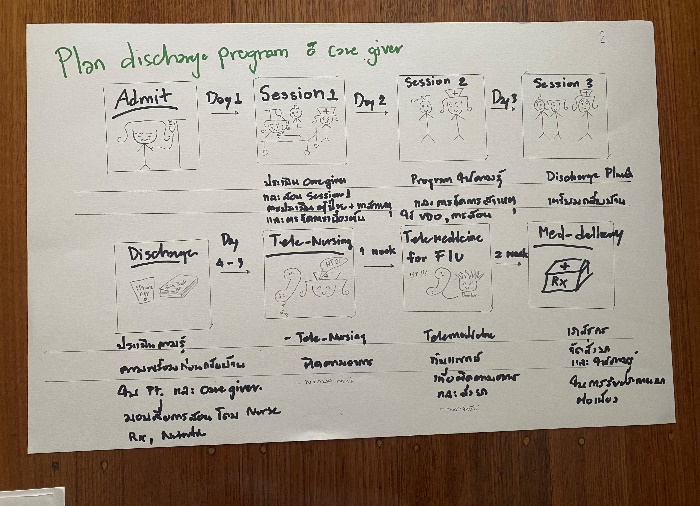 | 1. A carer plays a significant role in chronic illness, influencing their decision-making and disease control. 2. Reduce costs and workload. |
| Telehealth CHF | Establishing teamwork pathway for individual care plan for support knowledge and self-management skills | Collaborative work of a multidisciplinary CHF team for develop an individual care plan which includes a holistic approach | **In-hospital**   1. Nurse ward coordinating with CHF case manager within 24 hours of admission. 2. First visit on admission day within 24 hours. 3. A multidisciplinary CHF teamwork collaborate and consult for individual case via Line application and continuity support for follow-up beyond two weeks.   **Outpatient remote monitoring provide by case manager:**   1. Follow-up at outpatient clinic at 2 weeks 2. Available 24/7 hr/day for provide the opportunity to contact if needed. 3. Telephone for remind patients for the next appointment. 4. Telephone education reinforcement every 3 months 5. Consultation with a multidisciplinary CHF team for treatment up titration or change as required. 6. Coordination with home healthcare for provide home visit for high-risk cases.   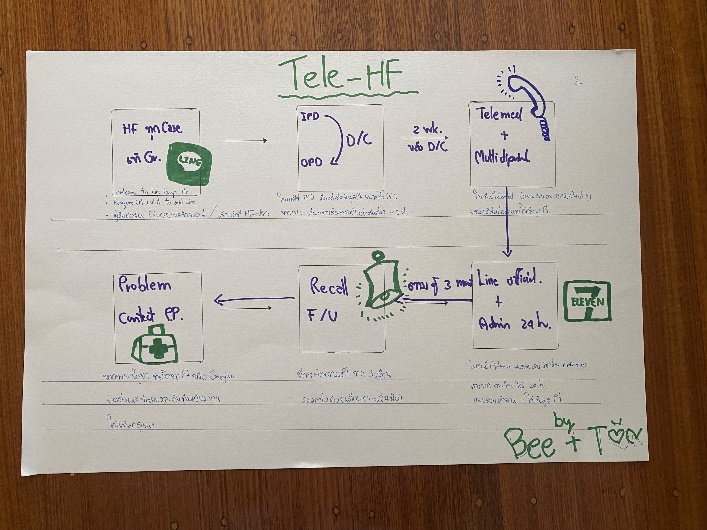 | 1. Challenges of assessment and follow-up via Line application. 2. Reduce costs and staff. 3. Provide the opportunity for access healthcare and ongoing care services. 4. It seems feasible and viable within a local context. |
| Family Home Health Care | Community resources support to encouraging patients and family to participate in effective community resources | Comprehensive discharge planning at early stages of re-admission and coordination with community support services | **In-hospital provide by case manager:**   1. First visit for providing CHF education and develop individual care plan with patients and caregivers. 2. Second visit within 24 hours before discharge for provide self-management education about dietary therapy, medication adherence, exercise and signs and symptoms of CHF deterioration by using teach back method.   **Discharge and outpatient support program provide by case manager:**   1. Peer support to patients with CHF by sharing experiences and provide opportunity to connect with like-minded people on the first follow-up appointment. 2. Refer to community-based services for provide home visit. 3. Telephone education reinforcement after discharge at day 3, 7, and 14. 4. Clinic visit at the first month then every 3 months.   **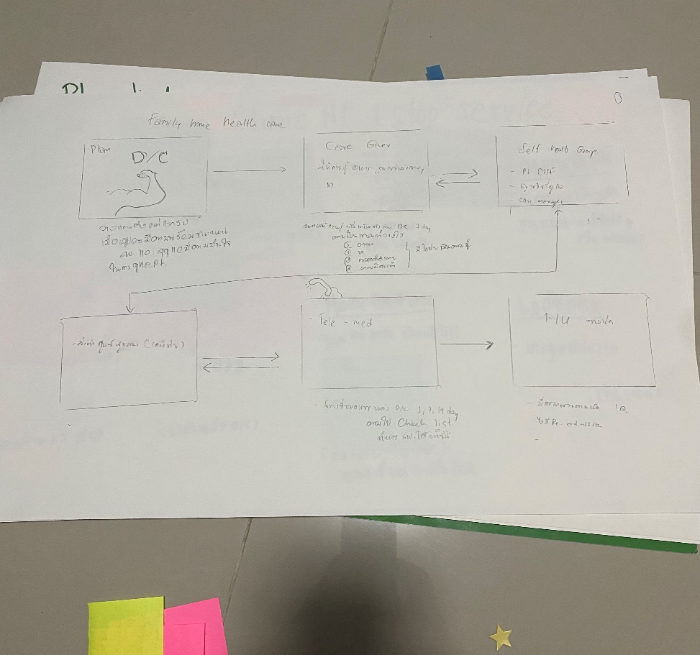** | 1. Peer support groups may assist patients improve their knowledge, self-management abilities, and quality of life. 2. May create workload problems for healthcare providers. 3. Due to limited facilities and resources, this implementation plan may not be practicable within a local environment. |
| Clinic CHF 1-stop Service | Establishing the CHF clinic. Organisation of healthcare support improvement at all level and perform CHF team for driven care. | Clinicians provide a final diagnosis and identify types and class of CHF. | **In-hospital**   1. Clinician provides the information about CHF, treatment and self-management. 2. Nurses provide education and materials using teach-back methods before discharge and consult CHF team. 3. Consultation with multidisciplinary CHF team (pharmacist, dietitians, physiotherapist, home healthcare) before discharge   **Outpatient disease self-management service.**   1. CHF team coordinated with home healthcare units for phone support or home visits for reinforce education within 7 days after discharge and then weekly. Summaries of each visit and provide message back to clinicians 2. Visit at CHF clinic within 14 days of discharge.   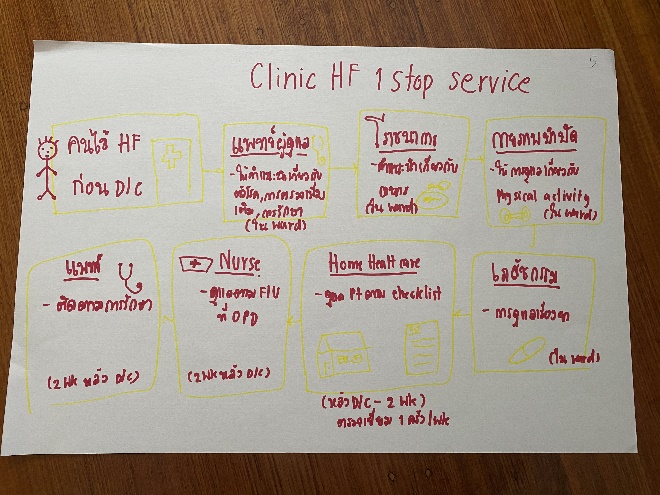 | 1. Organisational redesign challenges and the formation of a multidisciplinary CHF team 2. This implantation plan is one of the highest priorities of demands and is viable to implement. 3. Building an CHF clinic is difficult due to limited facilities and funding. 4. Due to limited facilities and resources, it is impossible to provide a home visit every week. 5. CHF clinics encourage equal collaboration in a multidisciplinary CHF team. |
| CHF clinic and home visit | Establishing CHF clinic and support actions for involvement of multidisciplinary CHF team | Implementation of structure follow-up and optimal clinic staffing models | **In-hospital**   1. Transfer CHF patients and caregiver to a CHF clinic before discharge to receive the self-management education and optimisation of CHF medications and structure of follow-up.   **Discharge and outpatient support program**   1. Follow-up at CHF clinic at 1 week after discharge 2. Access ongoing care and consultation support by a multidisciplinary CHF team via Line application within 48 hours 3. Coordinate with community services within 2 weeks after being discharge and then weekly. 4. Additional support such as psychologically   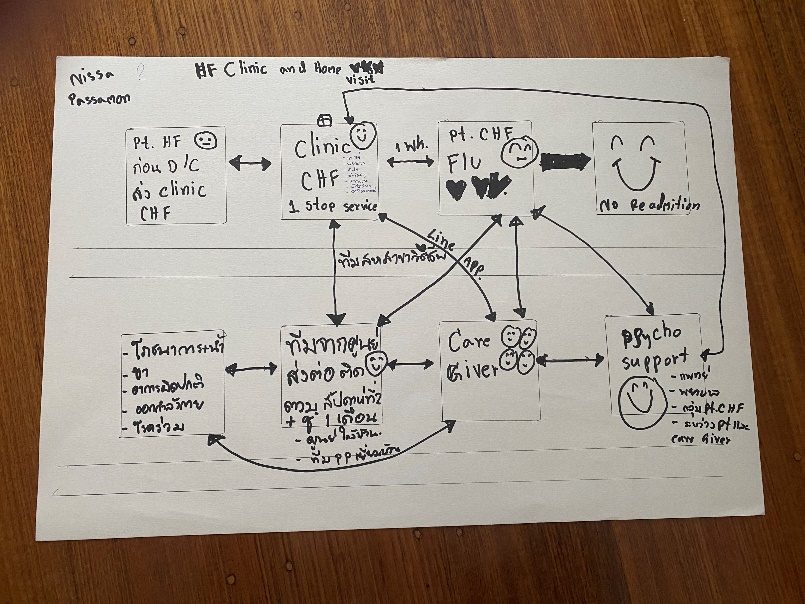 | 1. The team proposed that this implantation plan include discharge planning at least once before transferring patients to an CHF clinic. 2. Enhance community involvement and improving patient safety. |
